# Hiding in Plain Sight: Evidence of Echeneidae Cloacal and Gill Diving Behavior in Manta Ray Hosts

**DOI:** 10.1002/ece3.73548

**Published:** 2026-05-11

**Authors:** Emily A. Yeager, Jessica Pate, Guy M. W. Stevens, Bryant Turffs, Catherine Macdonald

**Affiliations:** ^1^ Department of Environmental Science and Policy, Rosenstiel School of Marine, Atmospheric, and Earth Science University of Miami Miami Florida USA; ^2^ Shark Research and Conservation Program, Rosenstiel School of Marine, Atmospheric, and Earth Science University of Miami Miami Florida USA; ^3^ Marine Megafauna Foundation West Palm Beach Florida USA; ^4^ The Manta Trust Catemwood House, Norwood Lane Dorset UK; ^5^ Maldives Manta Conservation Programme Malé Republic of the Maldives

**Keywords:** behavior, remora, symbiosis, symbiosis continuum

## Abstract

Symbioses between remoras (Family Echeneidae) and marine megafauna are well‐documented across diverse lineages. However, despite recent advancements in understanding the intricacies of these interactions, the dynamics of these relationships remain poorly understood, largely due to the highly mobile nature of both host and symbiont. Here we report seven observations of Echeneidae cloacal diving behavior in manta rays. These observations span all three currently described species of manta rays (*Mobula yarae, Mobula birostris*, and *Mobula alfredi*), demonstrate that large Echeneidae can perform cloacal diving behavior in both juvenile and adult manta rays, and show that this behavior occurs across multiple ocean basins. We also document one observation of Echeneidae attachment beneath a host's gill slit and several occurrences of gill injuries consistent with Echeneidae intrusion. These observations contribute to the growing database of Echeneidae‐host behavioral interactions and provide an important foundation for understanding the extent, diversity, and dynamics underlying these highly debated, cryptic megafauna‐symbiont interactions in marine environments. By providing new evidence of the complexity of symbiotic relationships in marine environments, this study also offers a multi‐species natural history context that may inform future research and conservation considerations.

## Graphical abstract

Here we document seven observations of Echeneidae cloacal diving behavior in manta rays and one observation of gill attachment behavior. These observations span all three currently described species of manta rays (*Mobula yarae, Mobula birostris*, and *Mobula alfredi*), demonstrate the ability of large Echeneidae to perform cloacal diving behavior in both juvenile and adult manta rays, showcase the behavior across several ocean basins, and provide an important foundation for understanding the extent, breadth, and dynamics underlying highly debated, cryptic, megafauna‐symbiont interactions in marine environments.

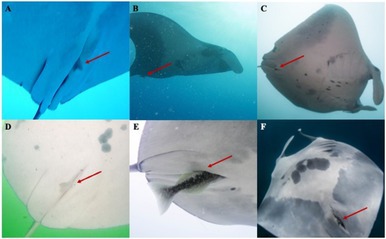

## 1 Introduction

Despite being well‐known for associating with a wide array of transient marine megafauna, the scope of, and interactions underlying host relationships with fish in the family Echeneidae remain largely understudied (Flammang et al. 2020; Gayford 2024; Nicholson‐Jack et al. 2021; Sazima and Grossman 2006). Family Echeneidae (ray‐finned fish commonly referred to as remoras, shark suckers, or suckerfish) are distributed globally throughout tropical and subtropical waters (Fertl and Landry Jr. 2009; Gayford 2024). Echeneidae have a specially modified dorsal adhesive disc that allows them to physically attach to a host using friction and vacuum‐like suction created by mobile pectinated lamellae (Britz and Johnson 2012; Wang et al. 2017). This adhesive behavior allows echeneid fish to ‘hitchhike’ on their hosts, actively facilitating their ability to travel long distances, including moving with hosts into environments they were not previously assumed to be physiologically adapted to, like the deep sea (Fontes et al. 2023; Gayford 2024; Nicholson‐Jack et al. 2021).

In addition to transport, associations with larger‐bodied hosts are theorized to facilitate commensalistic—namely phoresy—and mutualistic behaviors where hitchhikers clean their hosts of parasites and benefit from consuming host prey scraps and fecal material (Castellano‐Gonzalez et al. 2025; Cressey and Lachner 1970; Dove and Robinson 2021; Gayford 2024; Nicholson‐Jack et al. 2021; Peterson et al. 2022). Yet, despite these commensalistic and mutualistic attributes, the true nature of Echeneidae‐host symbiosis remains widely debated. Notably, Echeneidae attachment can cause physical injury to the host, can increase hydrodynamic drag, and can negatively influence overall host energy demand, especially when present in significant numbers or at larger sizes (Flammang et al. 2020; Gayford 2024; Kale et al. 2025; Strike et al. 2022). As hosts have limited ability to disengage hitchhikers and can be negatively affected by their presence, researchers increasingly argue that Echeneidae symbiosis may have more parasitic tendencies, indicating that this symbiosis is dynamic and likely exists on a continuum between mutualism and parasitism (Brunnschweiler et al. 2020; Gayford 2024; Klimley et al. 2024; Strike et al. 2022).

Previous research has demonstrated that the relationships between some Echeneidae species and their hosts are relatively stable and an important component of broader host‐associated fish aggregation composition (Castellano‐Gonzalez et al. 2025; Gayford 2024; Nicholson‐Jack et al. 2021; Shiffman et al. 2025; Yeager et al. 2026). For example, of the four families of fish frequently observed aggregating with juvenile Atlantic manta rays (*Mobula yarae*) in the western Atlantic, Echeneidae are the most frequent and ecologically stable associate (Yeager et al. 2026). Research on other species of mobulid rays has emphasized the frequent association between rays and echeneid fish and hypothesized that these rays may serve as important habitats for their Echeneidae associates, such as reproductive habitat for mating pairs (Castellano‐Gonzalez et al. 2025; Stevens et al. 2025). In one study on sicklefin devil rays ( *Mobula tarapacana* ), 98.8% of devil rays observed had at least one associated echeneid fish and, generally, associated with an average of 2.45 ± 0.90 Echeneidae fish per observation (Castellano‐Gonzalez et al. 2025). Castellano‐Gonzalez et al. (2025) described *Remora* spp. as most frequently attaching to the anterior of devil rays with no dorsal/ventral preference. Alternatively, Yeager et al. (2026) detailed echeneid fish (Remora spp. and Echeneis spp.) preferentially associating with the ventral, anterior, left‐hand side of juvenile Atlantic manta rays. Despite variation in specific locality of Echeneidae on their host, both studies mention frequent Echeneidae associations with morphological features including their host's eyes, gills, and tail (Castellano‐Gonzalez et al. 2025; Yeager et al. 2026).

While echeneid fish are most extensively documented attaching to the external body of marine megafauna, in some large‐bodied hosts, Echeneidae symbionts have been observed entering semi‐internal structures (Dove and Robinson 2021; Green et al. 2023; Moulinie et al. 2026; Nicholson‐Jack et al. 2021). Green et al. 2023, for example, documented remoras ( *Remora remora* ) entering gill slits, cloacal openings, and spiracles on whale sharks ( *Rhincodon typus* ). There is also evidence of small echeneid fish entering the mouths of lemon sharks ( *Negaprion brevirostris* ; Mori et al. 2025) and remaining in the buccal cavities of adult reef manta rays ( *M. alfredi* ; Nicholson‐Jack et al. 2021; Stevens et al. 2018). One recent observation by Moulinie et al. (2026) documented a white suckerfish (*Remora albescens*) entering the cloaca of a reef manta ray. Hypotheses surrounding these intrusive echeneid behaviors suggest that small echeneid fish may physically enter their host's semi‐internal structures to facilitate cleaning behavior or for protection (Castellano‐Gonzalez et al. 2025; Mori et al. 2025; Stevens et al. 2018). However, limited observations of these cryptic behaviors impede scientific descriptions of the mechanisms driving their occurrence (Castellano‐Gonzalez et al. 2025; Dove and Robinson 2021; Green et al. 2023; Mori et al. 2025; Stevens et al. 2018).

This study aims to build upon these cryptic observations and to add evidence of intrusive Echeneidae behavior by collating opportunistic observations of Echeneidae‐host interactions collected by the Marine Megafauna Foundation and The Manta Trust during routine manta ray surveys. Utilizing opportunistic video and photographic evidence, this study documents echeneid fish entering manta ray cloacal openings (henceforth described as cloacal diving) in all three currently described species of manta rays. We also show evidence of echeneid fish attachment within manta ray gill slits and document gill injuries we hypothesize derived from prior echeneid attachment. Together, these opportunistic observations of cloacal diving and gill attachment behavior are the largest currently described dataset of Echeneidae attachment to semi‐internal structures in marine megafauna. These observations add important documentation to the growing knowledge base on Echeneidae‐host associations and offer insight into additional drivers that may explain the ecological dynamics underlying this cryptic symbiotic relationship.

## 2 Methods

Between 2010 and 2025, research teams led by the Marine Megafauna Foundation and The Manta Trust routinely conducted surveys at known manta ray aggregation sites including southeast Florida, Fuvahmulah and Baa Atolls in the Maldives, and Zavora, Mozambique. Across all surveys, a combination of drones, SCUBA, and freedivers were used to collect video and photographic data on manta rays. This visual documentation included ventral imagery of manta ray spot patterns (which are used to identify individual manta rays; Marshall et al. 2011) and sex (identified via the presence or absence of claspers) and imagery allowing assessment of manta ray size and health, but often varied significantly in length and scope depending on the survey. Manta ray age class was determined by either visual appearance (e.g., mantas with smaller wingspans or smaller claspers occurring in known nursery areas were characterized as juveniles) or by standardized laser measurements while manta ray health was characterized by the presence of injury or fishing gear. Over the course of this study period, the Marine Megafauna Foundation and the Manta Trust conducted thousands of individual manta ray surveys documenting the presence and health of Reef manta rays ( *M. alfredi* ), Atlantic manta rays (*M. yarae*), and Oceanic manta rays ( *M. birostris* ) across two ocean basins. On seven surveys (< 1% of total survey effort), researchers documented echeneid fish engaging in cloacal diving behavior. On one additional survey, an echeneid fish was observed actively attached to a host's gill arch.

As in recordings of cloacal diving behavior, manta rays were almost always only seen briefly in passing with echeneid fish already within their cloacal openings, and video and photographic quality varied greatly between 2010 and 2025, robust, species‐level identification of echeneid fish was not feasible. Thus, the common term remora was used to describe all Echeneidae, and species‐level identification was only made in one instance where the entire remora was visible prior to engaging in cloacal diving behavior. Where possible, the relative size of the remora compared to its manta host was described using the same parameters as Yeager et al. (2026) and species‐level identification was made to the best approximation using FishBase.

## 3 Behavioral Observations

**FIGURE 1:**
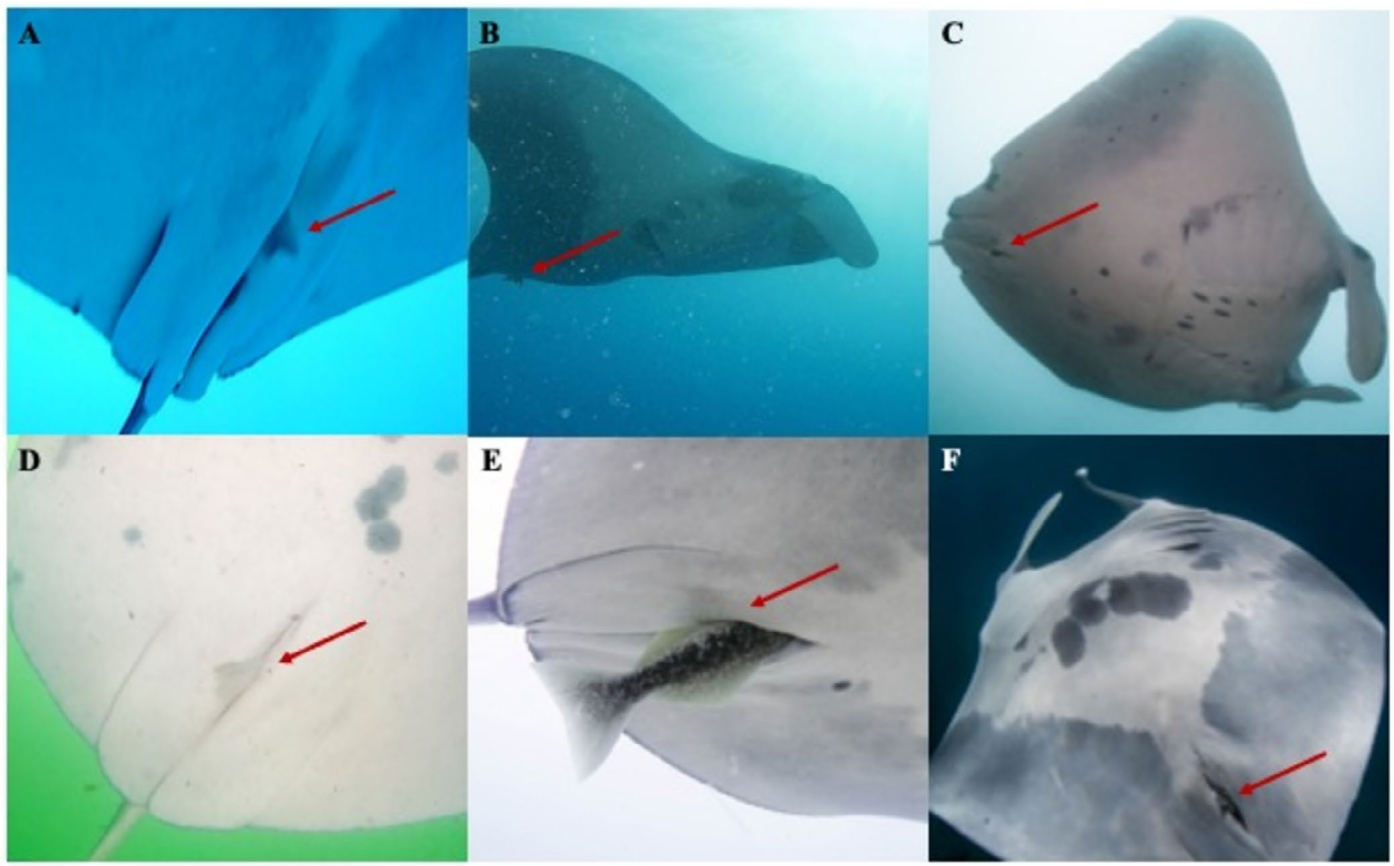
Photographs documenting the presence of remoras within manta ray cloacal openings. (A) March 2010 observation of a remora's tail within the cloacal opening of a *Mobula birostris* in the Maldives (Image Credit: Stefan Bersch). (B) June 2010 observation of a remora's tail protruding from a *Mobula birostris* in the Maldives (Image Credit: Lisa Allison). (C) September 15, 2017 observation of a remora's tail protruding from the cloacal opening of a *Mobula alfredi* in Mozambique (Image Credit: Anna Flam). (D) September 19, 2017 observation of a remora's tail within a *Mobula alfredi* in Mozambique (Image Credit: Anna Flam). (E) July 2021 observation of a *Remora remora* (identified via its posterior) protruding from a *Mobula yarae* cloacal opening in Florida, USA. (F) October 2025 observation of a remora's tail within a female *Mobula yarae* in Florida, USA. Red arrows indicate echeneid fish.

Seven observations of remora cloacal diving behavior and one observation of gill attachment were made between 2010 and 2025 (Table 1). Two of these observations occurred in the Maldives (both in 2010), two occurred in Mozambique (both in 2017), and three occurred in southeast Florida (2021–2025). Both observations of cloacal diving behavior in the Maldives documented interactions between remoras and Oceanic manta rays ( *M. birostris* ; Figure 1A,B) while those in Mozambique were between remoras and Reef manta rays ( *M. alfredi* ; Figure 1C,D). The three observations in southeast Florida occurred in a described nursery (Pate and Marshall 2020) and were all of remoras in the cloacal openings of Atlantic manta rays (*M. yarae;* Figure 1E,F and Video 1). As these observations occurred in adult and juvenile hosts, in both the Atlantic and Indian Oceans, and across all three currently described species of manta ray, they highlight that remora cloacal diving behavior in manta rays appears demographically, geographically, and taxonomically widespread.

**TABLE 1:** Observations of remora cloacal diving behavior during manta ray surveys from 2010 to 2025.

Date of observation	Location of observation	Manta ray species	Manta ray sex	Research group
March 2010	Fuvahmulah Atoll, Maldives	*M. birostris*	Male	The Manta Trust
June 2010	Fuvahmulah Atoll, Maldives	*M. birostris*	Unknown	The Manta Trust
15 September 2017	Red Sands, Zavora, Mozambique	*M. alfredi*	Female	Marine Megafauna Foundation
17 September 2017	Red Sands, Zavora, Mozambique	*M. alfredi*	Female	Marine Megafauna Foundation
10 July 2021	Florida, USA	*M. yarae*	Male	MMF Florida Manta Project
13 July 2023^a^	Florida, USA	*M. yarae*	Male	MMF Florida Manta Project
28 October 2025	Florida USA	*M. yarae*	Female	MMF Florida Manta Project

^a^ Indicates video of behavior provided.

**VIDEO 1:** Video of a *Remora remora* entering a *Mobula yarae* cloacal opening in Florida, USA. (This figure is also available on FigShare: 10.6084/m9.figshare.31828684). Video content can be viewed at https://onlinelibrary.wiley.com/doi/10.1002/ece3.73548.

Despite the scope of these observations, photographic evidence alone fails to capture the drivers behind the occurrence of this intrusive behavior. One observation of cloacal diving behavior from July 13, 2023, that was caught on video helps to provide some additional context (Video 1). In this observation, a freediver approached an adult Atlantic manta ray from behind. Upon initial approach, a medium‐sized *Remora remora* was visible near the manta ray's pelvic fins. Once the diver passed into the ventral plane of the manta ray, the remora appeared to startle and quickly inserted itself into the manta ray's cloacal opening. In response to this intrusion, the manta ray briefly shuddered before continuing to swim away with the remora still inside of its cloacal opening. While this video clip was brief and does not show the remora reemerging from the cloacal opening, it is possible the unexpected presence of the freediver prompted the cloacal diving behavior, indicating the possibility that this behavior could be a response to perceived predation risk or other threat by the remora.

**FIGURE 2:**
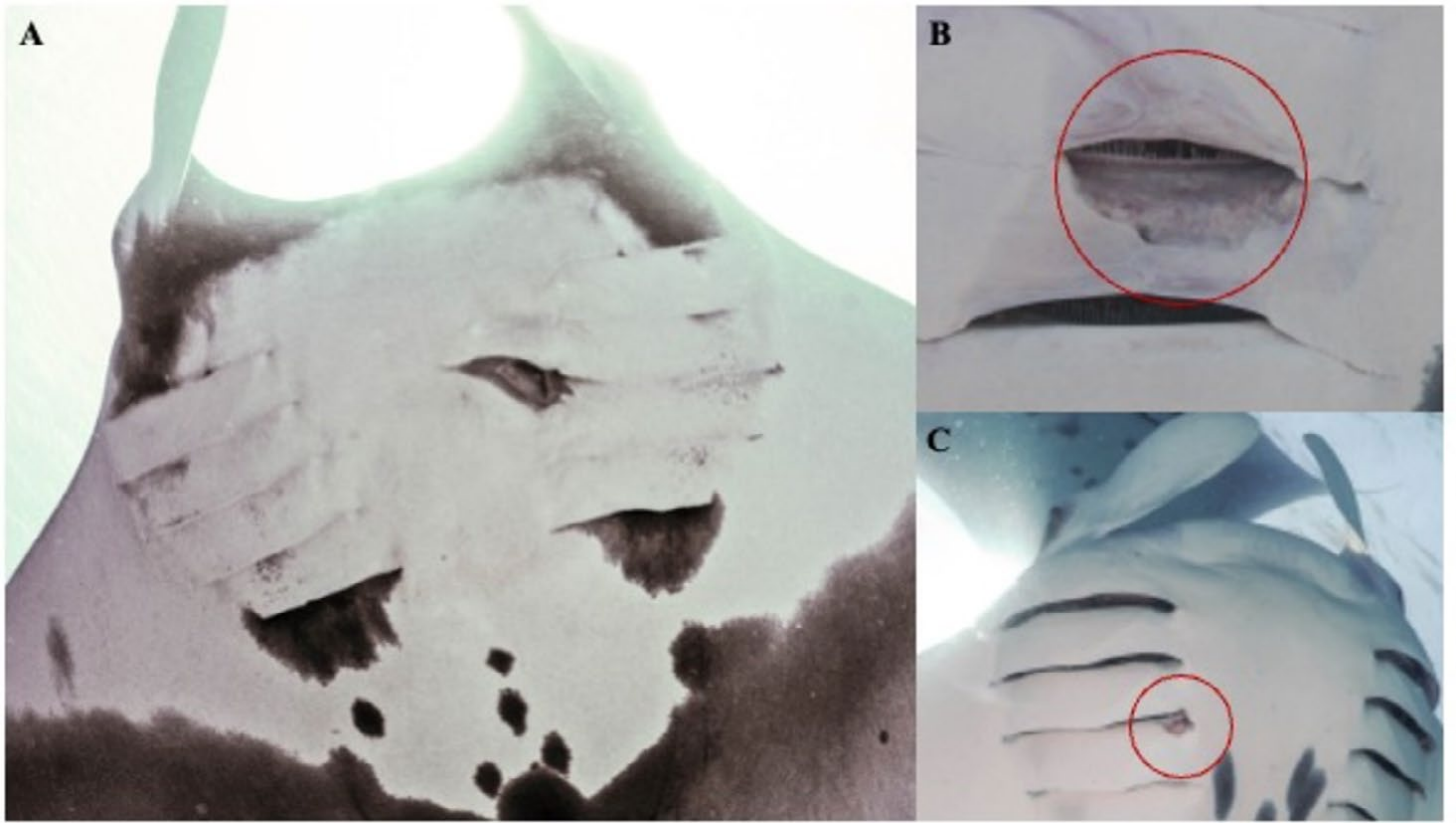
Photographic evidence of remora presence and perceived gill injury to manta ray gill slits. (A) February 20, 2011 observation of a *Remora remora* within a *M. birostris* gill slit in Fuvahmulah Atoll, Maldives. (B) February 11, 2010 observation of *M. alfredi* gill slit injury at Baa Atoll, Maldives. (C) August 29, 2011 observation of *M. alfredi* gill slit injury at Baa Atoll, Maldives. Red arrow indicates an echeneid fish, red circle indicates a gill injury. Image credits: Guy Stevens.

In addition to remora cloacal diving, divers also documented evidence of remora attachment to host gills. While this is not unexpected, as remoras have been repeatedly documented attaching near or on the external gill flaps of manta rays, physical intrusion into the inner gill slits has not been extensively recorded (Castellano‐Gonzalez et al. 2025; Yeager et al. 2026). On February 20, 2011, at Fuvahmulah Atoll in the Maldives, divers documented a remora in the gill slit of an injured Oceanic manta ray ( *M. birostris* ; Figure 2A). The remora appeared to be deeply embedded in the gill arch of the manta ray as only its tail was visible. While researchers were only able to record this behavior once, several other manta rays ( *M. alfredi* ) at Baa Atoll, Maldives were observed with similar injuries to their gills which could have also been caused by previous remora gill intrusion (Figure 2B,C). While it is not possible to definitively confirm that these injuries were caused by remora attachment, as other injury drivers such as parasite abrasion or mechanical damage are possible, remoras have previously been hypothesized to cause similar injuries on manta ray hosts (Strike et al. 2022). Evidence from non‐manta ray hosts, including cetaceans and sharks, also indicates that remoras can cause physical injury through hitchhiking behavior (Fertl and Landry 2018; C. Macdonald unpublished data).

## 4 Discussion

These observations of remora cloacal diving and gill attachment behaviors provide important insight into the extent of, and dynamics underlying, Echeneidae‐host symbiotic interactions. While this study documents seven observations of remora cloacal diving behavior and one observation of gill attachment (< 1% of total manta ray interactions), the subtlety of the images recorded (e.g., only the very end of the remora's tail protruding from the gill or cloacal opening; Figures 1 and 2) indicate that the frequency of this behavior could be underreported if visual indicators are missed or not obviously present. Despite extensive documentation of remoras associating with a wide array of marine megafauna, the nature of these relationships remains debated (Brunnschweiler et al. 2020; Castellano‐Gonzalez et al. 2025; Gayford 2024). Evidence of mutualistic (e.g., host cleaning and remora feeding), commensalistic (e.g., remora phoresy), and parasitic (e.g., energetic costs to hosts carrying large remora loads) suggests that this symbiosis exists along a continuum from mutualism to parasitism and that interaction outcomes vary across contexts (Castellano‐Gonzalez et al. 2025; Cressey and Lachner 1970; Dove and Robinson 2021; Kale et al. 2025; Gayford 2024; Nicholson‐Jack et al. 2021; Peterson et al. 2022; Yeager et al. 2026). Although no single observation can resolve this debate, increased documentation of cryptic behaviors, such as those described here, can add meaningful context for the conditions under which remora‐host interactions may shift along this continuum.

While remora cloacal diving and gill attachment behavior has been previously documented in large‐bodied whale sharks, it has not been formally recorded in other, smaller‐bodied organisms where these intrusive interactions could have greater fitness costs to the host (Green et al. 2023). Researchers including Castellano‐Gonzalez et al. (2025) have hypothesized that cloacal diving and gill attachment likely occur in manta rays but, as the cloacal and gill openings are inherently smaller in mobulid rays than they are in larger species, these interactions would likely feature small echeneid fish. Therefore, it is notable that at least one interaction recorded (Video 1) in this study featured a medium‐sized remora which hypothetically would be more consequential to a host than a smaller fish. While this behavior was documented through short interactions and the amount of time a remora may spend inside a cloacal opening is unknown, the presence of a moderately‐sized remora in a manta ray's cloacal opening could impede mating behavior, live birth, or defecation if the cloacal diving behavior occurs for extensive periods of time. As (Nicholson‐Jack et al. 2021) documented increased associations between remoras ( *E. naucrates* ) and near‐term pregnant Reef manta rays ( *M. alfredi* ), it is plausible that cloacal diving associations could have reproductive effects.

Additional documentation of remora attachment inside manta ray gill slits supports arguments that remora associations could have negative costs to their hosts. Mobulid gills are highly complex and are equipped with unique filter pads and filtration systems (Paig‐Tran et al. 2013). Mechanical damage to the gill arch could therefore have long‐lasting impacts on manta ray fitness. While this study documents one occurrence of remora attachment deep within the host's gill arch and several observations of presumed remora‐derived gill injuries, due to the temporal extent of these observations, it is not possible to know definitively if the observed injuries were truly remora‐caused. Other sources, such as parasite intrusion or physical injury from fishing gear entanglement or predation are possible, but the localized nature of the injuries and previous observations of remora injuries in elasmobranchs and cetaceans hosts, suggest that remoras are likely responsible for these injuries (Fertl and Landry 2018; C. Macdonald unpublished data; Savage et al. 2026; Strike et al. 2022). Although this dataset is observational and cannot answer questions about direct physiological effects (e.g., respiration or stress) of cloacal diving and gill attachment behavior to manta ray hosts, further research into these areas are of interest in understanding the full scope of the effects of remoras on their hosts.

**FIGURE 3:**
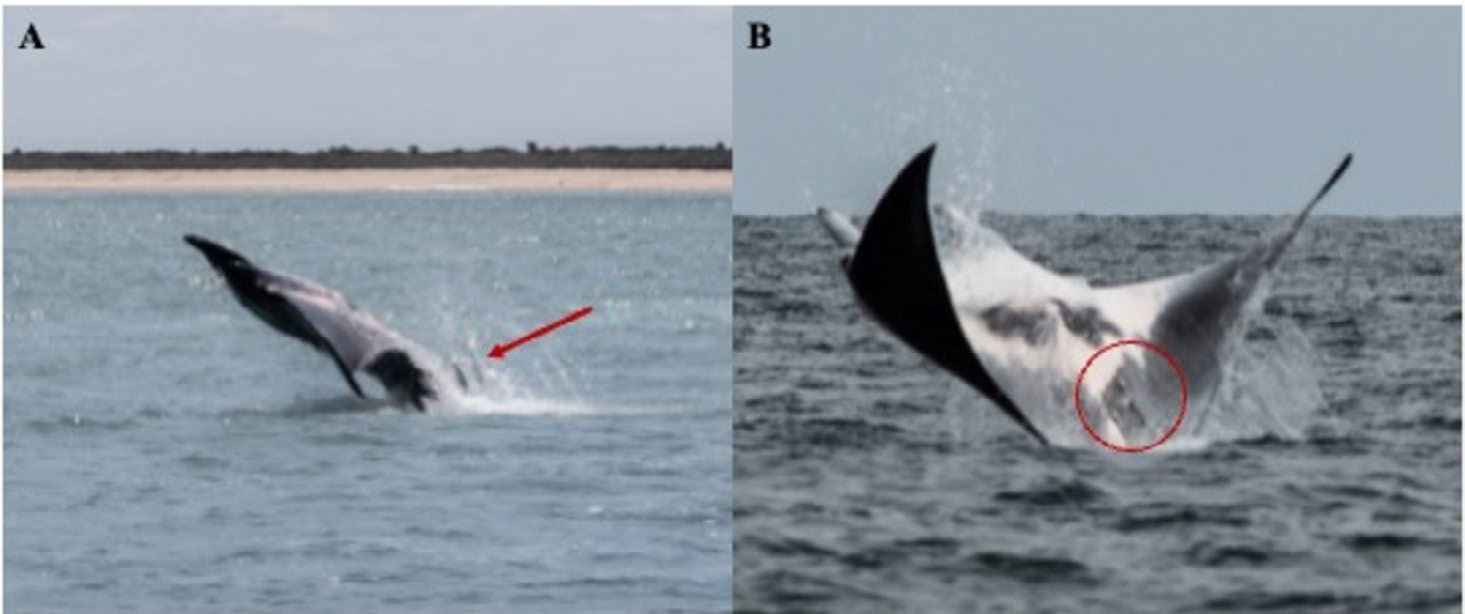
Photographs of remoras breeching alongside manta ray (*Mobula yarae*) hosts in central Florida. (A) Two remoras breaching alongside a manta ray, red arrow indicates remora presence. (B) One remora exiting the water with a manta host, red circle indicates remora presence. Image credits: Bryant Turffs.

Exploration of the potentially context‐driven parasitic nature of remora‐host symbioses is further supported by evidence of elasmobranch hosts attempting to remove or displace attached remoras (Homma et al. 1999; Ritter 2011). Both manta rays and sharks, for example, have been observed chafing against sandy and rocky‐bottom substrate to dislodge hitchhikers (Homma et al. 1999; Ritter 2011). Researchers with the Marine Megafauna Foundation have also observed manta rays flicking their pectoral fins and breaching, presumably to remove attached remoras (Figure 3). Yet, despite these removal efforts, manta rays still may not be able to fully dislodge remora hitchhikers which can quickly re‐attach after chafing or even co‐breach with their host (Figure 3; Klimley et al. 2024). Given that remora removal from the external body is challenging, symbiont removal from gill and cloacal openings may be even more difficult—especially as these openings can be more structurally sensitive (Paig‐Tran et al. 2013). While the reasons behind remora‐removal behavior can only be hypothesized, the multiple strategies for removal and the repeated documentation of removal attempts across hosts provide some evidence of host perception of remora parasitic tendencies.

Although remora cloacal diving and gill attachment behaviors could have negative effects for manta ray hosts, their benefit to echeneid fish is more apparent. Videographic documentation of cloacal diving behavior from July 2023 showed a remora entering a manta ray's cloaca upon the sudden appearance of a freediver (Video 1). This could indicate a predator‐avoidance response. Larger predators have been documented using manta ray aggregations as hunting grounds, suggesting that, despite associating with a larger organism, remoras may still be at predation risk (Brunnschweiler et al. 2020; Jason 2018; Knochel et al. 2023; Wedekin et al. 2004). One study on captive sailfish even hypothesized that remora presence in the gill cavity was likely a predator‐avoidance technique (Mori et al. 2025). Therefore, it is reasonable to hypothesize that cloacal diving behavior could serve as a similar avoidance technique.

Feeding behavior may also be relevant to remora associations with gill slits and cloacal openings. Echeneid fish have been documented consuming host fecal material across species and gills tend to contain parasitic copepods which remoras are also known to feed on (Castellano‐Gonzalez et al. 2025; Dove and Robinson 2021; Homma et al. 1999; A. Kusel, unpublished data; Nicholson‐Jack et al. 2021). While it is likely that predator avoidance and feeding play some role in the occurrence of cloacal diving and gill attachment behaviors, it is also possible that hydrodynamic factors could be influential (Castellano‐Gonzalez et al. 2025; Flammang et al. 2020). Previous work on blue whales ( *Balaenoptera musculus* ) and devil rays has shown preferential Echeneidae attachment to sites of reduced drag (Castellano‐Gonzalez et al. 2025; Flammang et al. 2020). As fully entering a host's cloaca or gill slit would likely substantially reduce remora‐associated drag, it is plausible that these behaviors could reduce the fitness costs of attaching to the exterior of a host's body.

Although the exact mechanisms driving cloacal diving and gill attachment behaviors remain unclear, it is apparent that Echeneidae‐host relationships are more physiologically and ecologically complex than previously understood. Remoras associate with marine organisms ranging from small teleosts to large mammals and appear to have somewhat stable ecological dynamics across host species and sizes (Flammang et al. 2020; Gayford 2024; Nicholson‐Jack et al. 2021; Shiffman et al. 2025; Yeager et al. 2026). Echeneidae symbionts are most often observed hitchhiking or participating in cleaning and feeding behaviors and can be attached to their host or free‐swimming alongside them (Castellano‐Gonzalez et al. 2025; Gayford 2024; Nicholson‐Jack et al. 2021; Yeager et al. 2026). Yet, evidence of cryptic harmful behaviors in new species indicates that these symbioses may be more parasitic, at least in some contexts, than previously understood. The observations presented here expand current understanding of cloacal diving and gill attachment behavior across host‐symbiont interactions and demonstrate that this behavior is conserved across ocean basins, in all currently described species of manta rays, and amongst manta rays of different sizes and age classes. Taken together with other observations of Echeneidae‐manta ray symbiotic interactions, these observations corroborate the likelihood that manta rays serve as mobile ecosystems for teleost fish and offer important insights into the dynamics underlying and driving, and the nature of, remora‐host symbioses.

## Author Contributions

**Emily A. Yeager:** conceptualization (equal), formal analysis (equal), investigation (equal), project administration (equal), visualization (equal), writing – original draft (lead), writing – review and editing (lead). **Jessica Pate:** conceptualization (equal), data curation (lead), funding acquisition (equal), investigation (supporting), project administration (equal), resources (equal), supervision (supporting), visualization (supporting), writing – review and editing (equal). **Guy M. W. Stevens:** data curation (equal), resources (supporting), validation (equal), visualization (equal), writing – review and editing (equal). **Bryant Turffs:** visualization (lead), writing – review and editing (equal). **Catherine Macdonald:** funding acquisition (equal), resources (equal), supervision (lead), writing – review and editing (equal).

## Ethics Statement

All fieldwork in this study was conducted by the Marine Megafauna Foundation's Florida Manta Project under FWC Special Activity License SAL‐25‐2053‐SRP and The Manta Trust. The Manta Trust carried out research under permit from the Maldives' government (annually renewable permit: PA/2020/PSR‐M07). No animal handling or collection of any biological samples was conducted.

## Conflicts of Interest

The authors declare no conflicts of interest.

## Data Availability

All images used for analysis in this manuscript are provided in the manuscript body and figures. Video 1 (the video of remora cloacal intrusion) can also be found on FigShare (10.6084/m9.figshare.31828684). For further information, please reach out to the corresponding author.
